# [18F]Florbetapir positron emission tomography: identification of muscle amyloid in inclusion body myositis and differentiation from polymyositis

**DOI:** 10.1136/annrheumdis-2018-214644

**Published:** 2019-02-13

**Authors:** James B Lilleker, Richard Hodgson, Mark Roberts, Karl Herholz, James Howard, Rainer Hinz, Hector Chinoy

**Affiliations:** 1 Centre for Musculoskeletal Research, School of Biological Sciences, Faculty of Biology, Medicine and Health, Manchester Academic Health Science Centre, University of Manchester, Manchester, UK; 2 Manchester Centre for Clinical Neuroscience, Salford Royal NHS Foundation Trust, Salford, UK; 3 Radiology Department, Salford Royal NHS Foundation Trust, Salford, United Kingdom; 4 The National Institute for Health Research Manchester Musculoskeletal Biomedical Research Centre, Manchester University Hospitals NHS FoundationTrust, Manchester Academic Health Science Centre, University of Manchester, Manchester, United Kingdom; 5 Wolfson Molecular Imaging Centre, University of Manchester, Manchester, UK; 6 Rheumatology Department, Salford Royal NHS Foundation Trust, Salford, United Kingdom

**Keywords:** inclusion body myositis, polymyositis, amyloid, positron emission tomography, diagnostics

## Abstract

**Objectives:**

With the tools available currently, confirming the diagnosis of inclusion body myositis (IBM) can be difficult. Many patients are initially misdiagnosed with polymyositis (PM). In this observational study at a UK adult neuromuscular centre, we investigated whether amyloid positron emission tomography could differentiate between IBM and PM.

**Methods:**

Ten patients with IBM and six with PM underwent clinical review, [18F]florbetapir positron emission tomography and MRI of skeletal musculature. Differences in [18F]florbetapir standardised uptake value ratios in skeletal muscle regions of interest were evaluated. Relationships between [18F]florbetapir standardised uptake value ratios and measures of disease severity (clinical and by MRI of skeletal muscle) were assessed.

**Results:**

[18F]florbetapir standardised uptake value ratios were significantly higher in those with IBM compared with PM for all assessed regions (total-[18F]florbetapir standardised uptake value ratio 1.45 (1.28 to 2.05) vs 1.01 (0.80 to 1.22), p=0.005). For total-[18F]florbetapir standardised uptake value ratios≥1.28, sensitivity and specificity for IBM was 80% and 100%, respectively.

**Conclusions:**

[18F]florbetapir amyloid positron emission tomography differentiates IBM from PM. Successful development could facilitate accurate diagnosis, inclusion in clinical trials and help avoid unnecessary exposure to potentially harmful treatments.

**Key messages**

**What is already known about this subject?**

- Positron emission tomography can detect tissue deposits of amyloid, potentially allowing non-invasive differentiation of inclusion body myositis (IBM) from polymyositis (PM).

**What does this study add?**

- Significantly increased intramuscular amyloid levels were found in IBM.
- Amyloid levels generally correlated poorly with disease severity, muscle inflammation and fatty infiltration levels.

**How might this impact on clinical practice or future developments?**

- Muscle amyloid imaging can differentiate between IBM and PM and could prove a useful future diagnostic modality.

## Introduction

Inclusion body myositis (IBM) is an acquired muscle disease with a slowly progressive course, culminating in severe disability.^1^ IBM is categorised as an inflammatory myopathy and shares histopathological features with polymyositis (PM), but immunosuppression does not modify progression.^2^ IBM is often diagnosed late and is commonly misdiagnosed initially as PM, due in part because differentiation on histopathological grounds can be difficult. In one study, five of nine patients with a diagnosis of ‘PM’ developed clinical features of IBM during follow-up, with such patients receiving unnecessary and potentially harmful immunosuppressive treatments.^3^

The presence of intramuscular beta-amyloid forms part of several IBM diagnostic criteria and is a key difference from PM.^4^ While this feature has a high diagnostic specificity, a relatively low sensitivity has been demonstrated, particularly in early IBM.^5^ Recent diagnostic criteria for IBM have shifted towards identification of the characteristic pattern of muscle weakness, with less strict histopathological requirements.^4^ While this has improved sensitivity, clinically detectable weakness implies that significant and irreversible muscle damage has occurred, reducing the likelihood that novel treatments will be effective.

We hypothesise that using amyloid positron emission tomography (amyloid-PET) to detect beta-amyloid within muscle can distinguish IBM from other inflammatory myopathies. Unlike muscle biopsy, imaging is non-invasive and large volumes of muscle can be studied, potentially improving sensitivity and facilitating earlier diagnosis. In this imaging study we compared the intramuscular amyloid burden, as determined using amyloid-PET, between IBM and PM. (E)-4-(2-(6-(2-(2-(2-18F-fluoroethoxy)ethoxy)ethoxy)pyridin-3-yl)vinyl)-N-methyl benzenamine, here referred to as [18F]florbetapir, was used as the amyloid imaging agent.^6 7^

## Methods

### Participants

Between October 2015 and October 2016, written informed consent was provided by 10 cases with IBM and 6 with PM selected from the database of patients attending the adult neuromuscular service at Salford Royal NHS Foundation Trust, UK. For the PM cohort, we restricted recruitment to those aged >45 years (online supplementary appendix section 3). IBM cases met European Neuromuscular Centre 2011 diagnostic criteria (‘clinicopathologically defined’ (n=8) or ‘clinically defined’ (n=2)).^8^ Those with PM met Bohan and Peter diagnostic criteria (probable or definite) and had a minimum classification probability of 75% using the International Myositis Classification Criteria Project criteria.^9–11^

10.1136/annrheumdis-2018-214644.supp1 Supplementary data

### Study procedures

#### Clinical outcomes

For those with IBM the Functional Rating Scale (IBM-FRS) was performed.^12^ In PM, the International Myositis Assessment & Clinical Studies Group disease activity core set measures were completed.^13^ Both groups had muscle strength assessed using the manual muscle testing 260 (MMT26) score and completed the Health Assessment Questionnaire disability index (HAQ-DI).^14^

#### PET

A target dose of 370 MBq (18F)florbetapir was administered by intravenous bolus. A CT scan from shoulders to ankles was performed using a Siemens Biograph TruePoint PET/CT camera for attenuation correction and definition of regions of interest (ROI).^15^ A PET emission scan of the same area commenced 45 min after radiotracer injection. Five minutes for each of the eight or nine bed positions was used, depending on subject height. PET images were reconstructed using 3D Ordered Subset Expectation Maximisation with three iterations and 21 subsets producing whole body images with almost isotropic voxels (2.6728 mm×2.6728 mm×2.027 mm) and a matrix size of 256×256 voxels per transaxial plane. A 3D Gaussian filter (full width at half maximum 3 mm) was applied postreconstruction to regularise images.

#### MRI

On the same day, whole body MRI was performed on a Philips Achieva 1.5 T scanner. A T1-weighted (TR 500 ms, TE 20 ms, bandwidth 220 Hz) sequence (to assess fatty infiltration of muscle) and a short tau inversion recovery (TR 5320 ms, TE 50 ms, TI 150 ms, bandwidth 170 Hz) sequence (to assess myoedema, a surrogate for muscle inflammation) were performed.

### Image processing

#### PET

Seven muscle ROIs were defined for each subject, consisting of all muscle within a 10 cm vertical stack of consecutive images from the anatomical CT scan. The placement of this section was centred on a slice 1/3 of the distance from the superior border of the patella to the anterior superior iliac spine for the thigh, 1/3 of the distance from the inferior border of the patella to the summit of the medial malleolus for the calf, 1/2 of the distance from the greater tuberosity of the humerus to the medial epicondyle for the left arm and 1/2 of the distance from the tip of the olecranon to the ulnar styloid process for the forearm. Each ROI was constructed using semiautomated threshold active contour segmentation tools within ITK-SNAP (online supplementary appendix section 1).^16^ Intensities of fat and muscle were specified (muscle: −10 to +100 HU; fat: −150 to −50 HU) and seed ‘bubbles’ placed within all visible musculature. Contour evolution could iterate until no further expansion of the ROI occurred.

For correction of non-specific radiotracer binding, a reference region was defined within the lumbar fat pad using the same centre landmark as the forearm ROI. Standardised [18F]florbetapir uptake values (SUVs) were calculated for each ROI by dividing the decay-corrected tissue mean concentration of radioactivity by the total injected radioactivity per body weight. Sum intensity means for all regions, upper limb regions and lower limb regions were calculated. SUV ratios (SUVRs) were calculated using the lumbar fat pad reference. This region was chosen as large volumes were available for selection and the location was easily matched between participants. Cerebral amyloid imaging studies have also shown increased statistical power when using lipid-rich reference regions.^17^ Given the lipophilic nature of florbetapir, it was assumed that tracer binding in the subcutaneous adipose was predominantly of the non-specific type.

#### MRI

Images were scored by a blinded musculoskeletal radiologist (JH) using semiquantitative scoring tools based on those in the literature.^18–20^ Severity of fatty infiltration (0: normal, 5: end-stage appearance) and extent of inflammatory change (0: normal, 5: entire muscle) were scored (online supplementary appendix section 2). For comparison with the amyloid-PET, mean fatty infiltration and inflammation scores for corresponding muscle regions were calculated.

### Statistical analysis

[18F]florbetapir SUVs and SUVRs for IBM were compared with PM using the Mann-Whitney Ranksum test in STATA for Windows V.13.0 (College Station, Texas, USA). For the IBM group, correlations between [18F]florbetapir SUVRs and clinical and MRI parameters of disease severity were examined using Spearman’s ranked correlation. Two-sided students t-test or Fisher’s exact test were used where appropriate. Receiver operating characteristic analysis was performed regarding the sensitivity and specificity of the total-[18F]florbetapir SUVRs for IBM. P<0.05 was considered as significant. Disease duration refers to the interval between diagnosis and the date of participation in the study.

### Ethical and regulatory approvals

The study was sponsored by the University of Manchester and authorised by the UK National Research Ethics Service (Greater Manchester West, 15/NW/0547) and the Administration of Radioactive Substances Advisory Committee (RPC number: 595/3586/33509).

## Results

**Figure 1:**
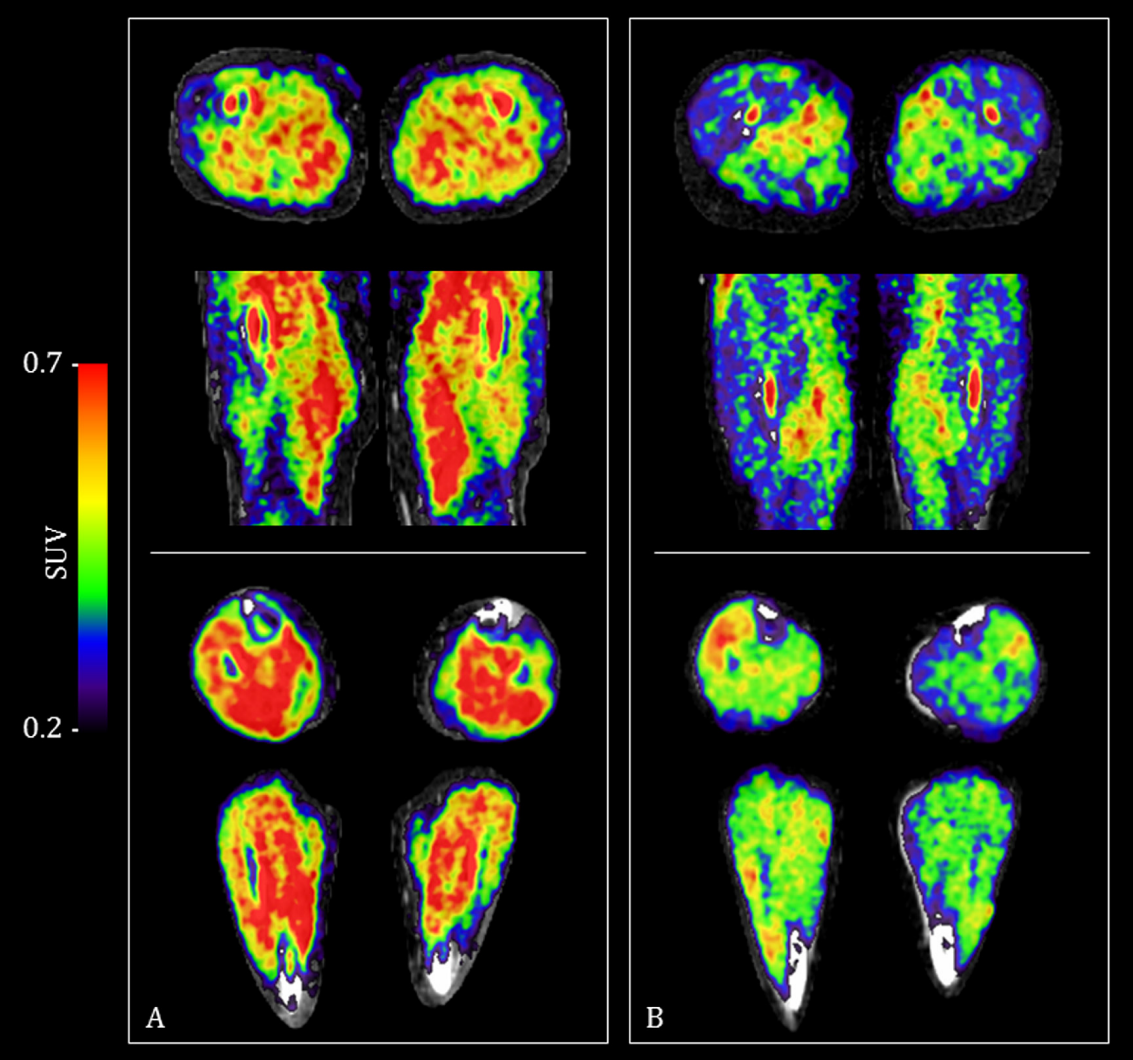
(18F)florbetapir PET/CT images showing differences in uptake between a participant with inclusion body myositis (panel A) and one with polymyositis (panel B). Increasing SUVs (red) indicate increased tracer uptake. [18F]Florbetapir PET images overlay spatially aligned CT images. Top of each panel depicts axial and coronal slices though the thigh. Bottom of each panel depicts axial and coronal slices though the calf. Each image is centred on the middle of the defined region of interest. PET, positron emission tomography; SUVs, standardised uptake values.

**Figure 2:**
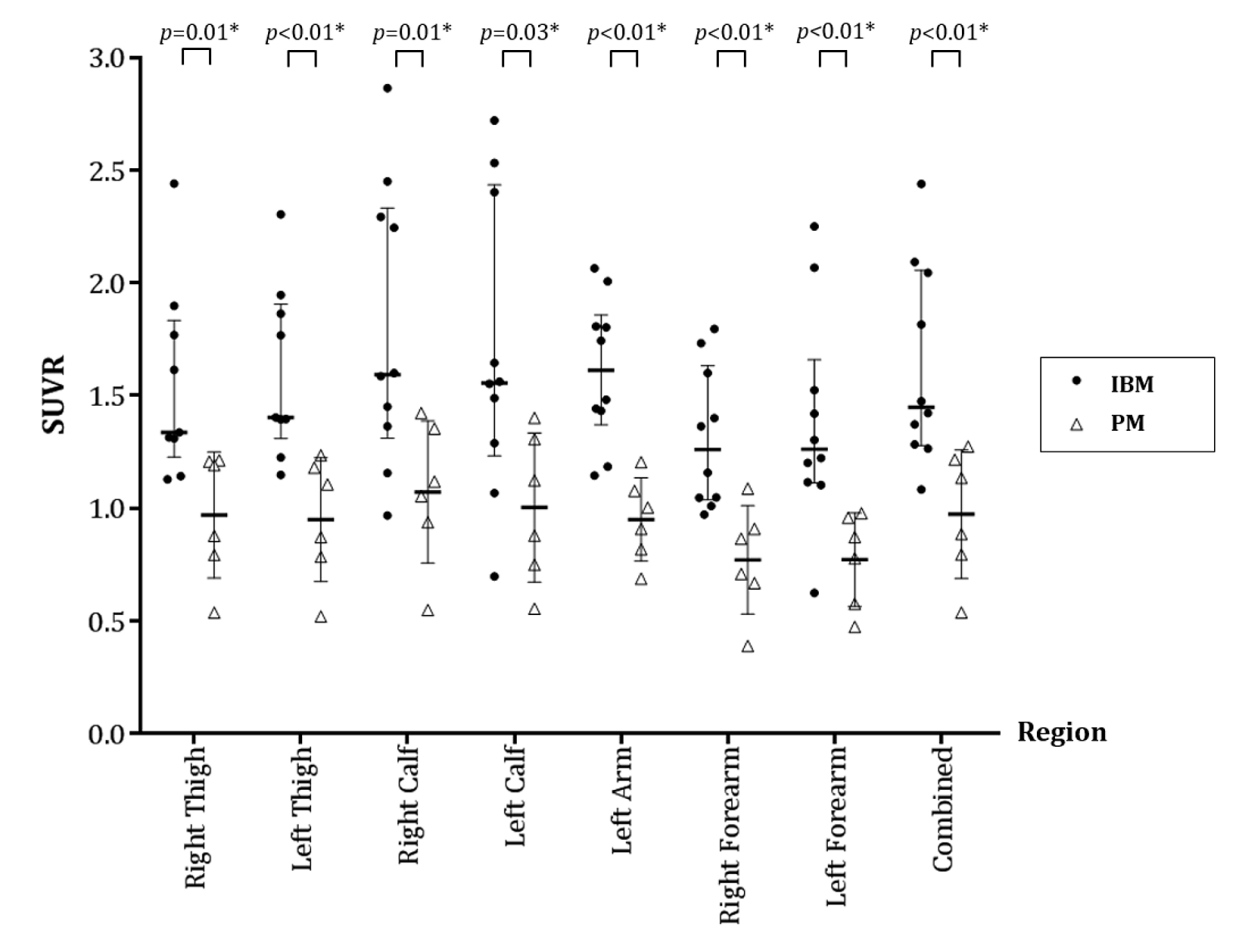
Comparison of SUVRs of [18F]florbetapir between participants with IBM (filled circles) and those with PM (open triangles) across seven different muscle regions and a combined region. Thick horizontal lines represent median SUVR and thin horizontal lines indicate the IQR. P values derived from Mann-Whitney Ranksum test. *Statistically significant difference (p<0.05). IBM, inclusion body myositis; PM, polymyositis; SUVR, standardised uptake value ratio.

Thirteen male and three female participants were studied (table 1). Three of the IBM group had previously received immunosuppressant medication, compared with all in the PM group. Visible differences were evident when comparing [18F]florbetapir PET/CT images between those with IBM and those with PM (figure 1). [18F]Florbetapir SUVRs were significantly higher in those with IBM for all ROIs (p value range 0.002–0.030) (table 1 and figure 2). For [18F]florbetapir SUVs (ie, without adjustment for non-specific radiotracer binding), only trends towards higher values in the IBM group were observed, except for the total-SUV region, where significantly higher values were also seen (table 1). For a total-[18F]florbetapir SUVR≥1.28 the diagnostic sensitivity for IBM was 80% and specificity 100% (area under curve 0.93).

**Table 1:** Clinical characteristics of subjects and muscle [18F]florbetapir uptake values

		IBM (n=10)	PM (n=6)	P value
Mean age in years at diagnosis (SD)		64.3 (8.4)	58.2 (10.7)	0.222*
Mean age in years at scan (SD)		68.3 (8.0)	59.7 (11.1)	0.092*
Mean disease duration at scan in years (SD)		4.0 (3.0)	1.5 (1.4)	0.079*
Gender (Male | Female)		9 | 1	4 | 2	**0.036**†
Mean manual muscle testing score (0–260) (SD)		236 (22.9)	256 (2.3)	0.052*
Mean Health Assessment Questionnaire disability index (SD)		1.3 (0.7)	0.8 (0.8)	0.192*
Mean IBM-Functional Rating Scale (0–40) (SD)		28.9 (5.3)	–	–
Mean physician global disease activity VAS (0–10) (SD)		–	1.8 (1.5)	–
Mean serum total creatine kinase level (IU/L) (SD)		579 (408)‡	308 (220)	–
Current immunosuppressive treatments (n)		Nil	Prednisolone (5/6) Methotrexate (2/6) Azathioprine (2/6) Cyclophosphamide (1/6)	–
Previous immunosuppressive treatments (n)		Prednisolone (3/10) Azathioprine (1/10) Mycophenolate (1/10)	Cyclophosphamide (2/6) Prednisolone (1/6) Mycophenolate (1/6) Azathioprine (1/6) Ciclosporin (1/6) IVIG (1/6)	–
Median [18F]florbetapir SUV (IQR)	Left arm	0.47 (0.41–0.55)	0.40 (0.36–0.48)	0.104§
	Right forearm	0.39 (0.35–0.42)	0.32 (0.27–0.40)	0.104§
	Left forearm	0.45 (0.32–0.55)	0.33 (0.30–0.36)	0.129§
	Right thigh¶	0.44 (0.43–0.52)	0.41 (0.37–0.45)	0.288§
	Left thigh¶	0.48 (0.43–0.51)	0.41 (0.36–0.45)	0.059§
	Right calf	0.51 (0.45–0.61)	0.46 (0.44–0.50)	0.233§
	Left calf	0.51 (0.40–0.58)	0.43 (0.39–0.45)	0.233§
	*Overall (total-SUV*)	0.48 (0.44–0.51)	0.42 (0.39–0.45)	**0.039§**
Median [18F]florbetapir SUVR (IQR)	Left arm¶	1.61 (1.43–1.81)	0.96 (0.82–1.08)	**0.002§**
	Right forearm	1.26 (1.05–1.60)	0.79 (0.67–0.91)	**0.005§**
	Left forearm	1.26 (1.12–1.52)	0.83 (0.58–0.96)	**0.005§**
	Right thigh**	1.34 (1.31–1.77)	1.04 (0.79–1.21)	**0.013§**
	Left thigh**	1.40 (1.40–1.87)	0.99 (0.79–1.18)	**0.005§**
	Right calf	1.59 (1.36–2.29)	1.09 (0.94–1.35)	**0.013§**
	Left calf	1.56 (1.29–2.40)	1.00 (0.75–1.31)	**0.030§**
	*Overall (total-SUVR*)	1.45 (1.28–2.05)	1.01 (0.80–1.22)	**0.005§**

Bold values indicate statistically significant differences.

*P values derive from two-sided students t-test.

†Fisher’s exact test.

‡For the IBM group, this refers to the peak serum creatine kinase level (it was not rechecked at the time of the scan).

§The Mann-Whitney Ranksum test.

¶The right arm was not used because radiotracer administration was via a venous cannula in the right antecubital fossa, except in two subjects (one with PM, one with IBM) where the reverse was true due to difficulties with cannula placement.

**n=9 for IBM group. Measurement in one subject could not be obtained due to very high levels of muscle atrophy and fatty replacement.

IBM, inclusion body myositis; IVIG, intravenous immunoglobulin; PM, polymyositis; SUV, standardised uptake value; SUVR, standardised uptake value ratio with reference region in lumbar fat pad; VAS, visual analogue scale.

In those with IBM, only in the calves were strong negative correlations between [18F]florbetapir SUVRs and muscle inflammation levels (by MRI) found (right calf Rho −0.73, p=0.02; left calf Rho −0.68, p=0.03). No significant correlation between [18F]florbetapir SUVRs and levels of fatty infiltration were identified. Furthermore, no significant relationships between the total-[18F]florbetapir SUVR and the age at scan, disease duration, MMT26, HAQ-DI or IBM-FRS were identified. This included subsets of the MMT26 and IBM-FRS restricted to upper limb and lower limb components compared with corresponding upper limb and lower limb [18F]florbetapir SUVRs (online supplementary appendix section 1 table 1). Amyloid deposits (by congo red staining) were only found in the diagnostic muscle biopsy of one IBM participant. No differences in the total-[18F]florbetapir SUVR were found according to the presence of degenerative biopsy features, including rimmed vacuoles (online supplementary appendix section 1 table 2).

## Discussion

In all assessed muscle groups, significantly increased [18F]florbetapir SUVRs were evident in IBM compared with PM. Sensitivity and specificity of the total-[18F]florbetapir SUVR for IBM was high, highlighting the potential diagnostic usefulness of muscle amyloid-PET. Further development of this technique could facilitate accurate diagnosis of IBM in those with early and otherwise undifferentiated disease, avoiding the use of potentially harmful treatments and facilitating inclusion in clinical trials.

To our knowledge, only one other published study used PET to detect intramuscular amyloid in IBM.^21^ Maetzler *et al* used the Pittsburgh-B (PiB) compound; a carbon-11 based radionucleotide with a half-life of approximately 20 min (compared with 110 min for fluorine-18), limiting its clinical use. Uniquely, we also performed same day muscle MRI and collected standardised clinical disease severity measures.

We used a semiautomated contour evolution method to select large sections of muscle for ROIs.^16^ It is likely that our method, rather than selecting small ellipsoid regions, produces more reliable results due to lower susceptibility to noise and bias from manual ROI placement. Borderline lower [18F]florbetapir SUVRs were found in the forearm when compared with other regions in both groups, potentially due to increased noise at the edge of the field of view. As we performed sequential exposures, comparison between different regions is susceptible to error, even after correction for radioactivity decay.

Our study is small and it is possible that factors other than diagnosis are confounding the results. A trend towards increased age at the time of scan is evident in the IBM group, but no significant correlations between age and the total-[18F]florbetapir SUVRs were evident (Rho=0.33, p=0.22), indicating that age alone is unlikely to explain the differences in intramuscular amyloid content between the groups. The IBM group also had borderline lower MMT26 scores. However, total-[18F]florbetapir SUVRs did not correlate significantly with measures of disease severity in this group, including the MMT26. Gender ratios are also different between the groups, but we are not aware of a clear rationale as to why this would independently influence the [18F]florbetapir SUVR.

This study has demonstrated the usefulness of muscle amyloid imaging using [18F]florbetapir PET in differentiating IBM from PM. By potentially improving the ability to accurately diagnose IBM, further development and validation of this technique could help to avoid the use of unnecessary medication and enhance involvement in clinical trials.
