# Supplementary material for: [18F]Florbetapir positron emission tomography: identification of muscle amyloid in inclusion body myositis and differentiation from polymyositis
Source: Ann Rheum Dis. 2019 Feb 13;78(5):657–62. doi: 10.1136/annrheumdis-2018-214644 (PMC6517800; doi:10.1136/annrheumdis-2018-214644)
Supplement: Supplementary data [file annrheumdis-2018-214644supp001.docx]

# Supplementary tables and figures

**Supplementary Figure 1:** Region of interest (ROI) definition in the thighs and calves using ITK-SNAP.(Yushkevich et al., 2006)

**
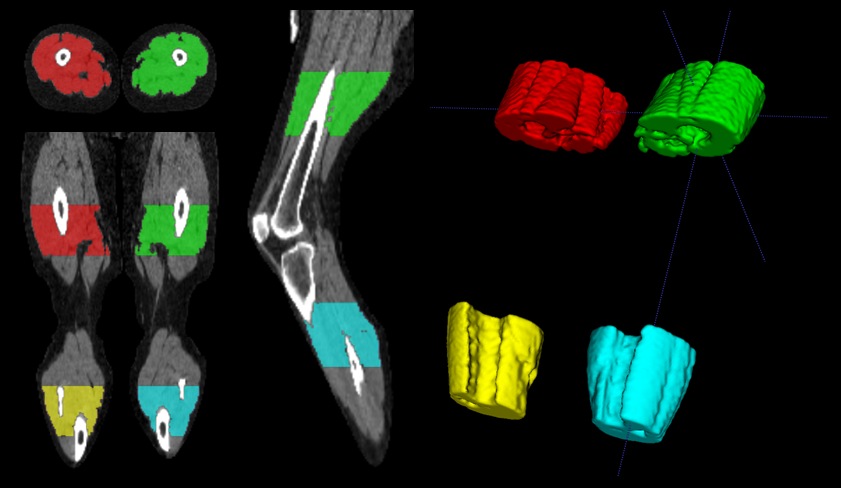
**

Left top = axial cross section at the level of the thigh. Left bottom = coronal section showing ROIs in the thigh and calf musculature. Middle = sagittal section showing ROIs in the thigh and calf musculature of the left leg. Right = 3D reconstruction of thigh and calf ROIs. Red = right thigh. Green = left thigh. Yellow = right calf. Cyan = left calf.

**Supplementary Table 1:**

Correlation of Magnetic Resonance Imaging measures of fatty infiltration and inflammation levels with
[18F]florbetapir standardised uptake value ratios in cases with inclusion body myositis (n=10)

| Region ([18F]florbetapir SUVR) | MRI Fatty infiltration level  Rho (p)* | MRI Inflammation level  Rho (p)* | Age at scan (years) Rho (p)* | Disease duration (years) Rho (p)* | HAQ-DI Rho (p)* | MMT26 Rho (p)* | IBM-FRS  Rho (p)* |
| --- | --- | --- | --- | --- | --- | --- | --- |
| Left Arm | 0.33 (0.35) | -0.30 (0.40) | - | - | - | 0.04 (0.91)** | -0.02 (0.96)** |
| Right Forearm | 0.14 (0.71) | -0.49 (0.15) | - | - | - |  |  |
| Left Forearm | -0.08 (0.82) | 0.15 (0.67) | - | - | - |  |  |
| Right thigh | 0.50 (0.17) | -0.12 (0.77) | - | - | - | 0.55 (0.10)** | 0.43 (0.21)** |
| Left thigh | -0.02 (0.97) | -0.22 (0.58) | - | - | - |  |  |
| Right calf | -0.02 (0.96) | **-0.73 (0.02)** | **-** | **-** | **-** |  |  |
| Left calf | 0.38 (0.27) | **-0.68 (0.03)** | **-** | **-** | **-** |  |  |
| Overall (total-SUVR) | 0.03 (0.93) | -0.52 (0.13) | -0.03 (0.93) | -0.36 (0.30) | -0.29 (0.43) | 0.37 (0.29) | 0.34 (0.34) |

*Spearman’s ranked correlation coefficient (Rho) between each imaging parameter and the corresponding [18F]florbetapir SUVRs for the same limb region.

**Subsets of MMT26 and IBM-FRS scores pertaining to relevant upper or lower limb domains.

Values in bold indicate statistically significant correlations. Disease duration refers to the interval between diagnosis and the date of participation in the study.

MRI = magnetic resonance imaging, HAQ-DI = health assessment questionnaire disability index, MMT-26 = manual muscle testing 26 score, IBM-FRS = inclusion body myositis functional rating scale, SUVR = standardised uptake value ratio (calculated using reference values derived from lumbar fat pad).

**Supplementary Table 2:**

Total [18F]florbetapir SUVR stratified according to diagnostic muscle biopsy findings in ten patients with Inclusion Body Myositis

| Muscle biopsy feature* | Present | Median total [18F]florbetapir SUVR (IQR) | Absent | Median total [18F]florbetapir SUVR (IQR) | Significance (ranksum test) |
| --- | --- | --- | --- | --- | --- |
| Rimmed Vacuoles | 8/10 (80%) | 1.45 (1.32, 2.07) | 2/10 (20%) | 1.55 (1.28, 1.82) | 0.794 |
| Amyloid deposits (by Congo Red) | 1/10 (10%) | 2.05 | 9/10 (90%) | 1.42 (1.28, 1.82) | 0.384 |
| TDP-43 (by immunofluorescence) | 6/7 (86%) | 1.73 (1.37, 2.09) | 1/7 (14%) | 1.27 | 0.317 |
| p62 (by immunofluorescence) | 3/4 (75%) | 1.82 (1.37, 2.44) | 1/4 (25%) | 1.28 | 0.180 |
| Tubulofilaments (by electron microscopy) | 1/1 (100%) | 1.48 | - | - | - |

*Not all features were assessed for on every biopsy.

IQR = interquartile range. P62 = Nucleoporin p62. SUVR = standardised uptake value ratio (calculated using reference values derived from lumbar fat pad). TDP-43 = transactive response DNA binding protein 43 kDa

# Imaging Definitions and Atlas

**In all cases, the following definitions also apply:**

| **Fatty infiltration score (using T1 sequence)** | |
| --- | --- |
| **Score**  Modified from (Mercuri et al., 2002) | **Interpretation (volume based assessment)** |
| 0 | Normal appearance |
| 1 | Very mild involvement: Early/trace moth-eaten appearance. Scattered small areas of increased signal. |
| 2 | Mild involvement: Late moth-eaten appearance with numerous discrete areas of increased signal with beginning confluence, comprising **less than 30% of the volume of the individual muscle** |
| 3 | Moderate involvement: Late moth-eaten appearance with numerous discrete areas of increased signal with beginning confluence, comprising **30-60% of the volume of the individual muscle** |
| 4 | Severe involvement: Washed-out appearance, fuzzy appearance due to confluent areas of increased signal. Muscle still present in the periphery. **(may be interpreted as 60-99% infiltration of volume of muscle with fat)** |
| 5 | End-stage appearance: Muscle replaced by increased signal connective tissue and fat, with only a rim of fascia and neurovascular structures distinguishable. **(may be interpreted as 100% replacement of muscle with fat)** |

NA = Not applicable (e.g. amputation, surgical resection)
NI = Not interpretable (e.g. artefact prevents reasonable interpretation)

| **Inflammation score (using STIR sequence)** | | | |
| --- | --- | --- | --- |
| **Visual image scoring system *modified* from Yao et al** (Yao & Gai, 2012) | ***Extent***  ***(volume based assessment)*** | ***Severity***  ***(on most affected slice)*** | |
| 0 | Normal | n/a | |
| 1 | Possible disease |  |  |
| 2 | Definite disease, < 30% of volume of muscle involved | A | Mild STIR hyper-intensity |
|  |  | B | Moderate-severe STIR hyper-intensity |
| 3 | Definite disease, 30-60% of volume of muscle involved | A | Mild STIR hyper-intensity |
|  |  | B | Moderate-severe STIR hyper-intensity |
| 4 | Definite disease, 60-99% of volume of muscle involved | A | Mild STIR hyper-intensity |
|  |  | B | Moderate-severe STIR hyper-intensity |
| 5 | Definite disease, Entirety of muscle involved | A | Mild STIR hyper-intensity |
|  |  | B | Moderate-severe STIR hyper-intensity |
|  | | **Definitions:**  **A** – Patchy/slight signal hyper-intensity – definitely brighter than normal muscle  **B** – More homogenous, bright or very bright signal, quality approaching that of fluid compartments (e.g. synovial fluid). | |

**Muscle MRI scoring *pro forma***

***Upper Limb***

| **Limb** | **Compart-ment** | **Muscle** | **Fatty infiltration score**  **(0-5)** | | **Atrophy score**  **(0-3)** | | **Inflammation score**  **(0-5, A/B)** | |
| --- | --- | --- | --- | --- | --- | --- | --- | --- |
|  |  |  | R | L | R | L | R | L |
| Shoulder Girdle | - | Trapezius |  |  |  |  |  |  |
|  |  | Deltoid |  |  |  |  |  |  |
|  |  | Supraspinatus |  |  |  |  |  |  |
|  |  | Infraspinatus |  |  |  |  |  |  |
|  |  | Subscapularis |  |  |  |  |  |  |
|  |  | Pec Major |  |  |  |  |  |  |
|  |  | Pec minor |  |  |  |  |  |  |
|  |  | Serratus anterior |  |  |  |  |  |  |
| Arm (muscles **not** assessed individually) | Anterior | Coracobrachialis, biceps, brachialis |  |  |  |  |  |  |
|  | Posterior | Triceps brachii, anconeus, articularis cubiti |  |  |  |  |  |  |
| Forearm (muscles  **not** assessed individually) | Anterior | Pronator teres, palmaris longus, flexor carpi radialis, flexor carpi ulnaris, flexor digitorum superficialis, pronator quadratus, flexor digitorum profundus, flexor pollicis longus |  |  |  |  |  |  |
|  | Posterior | Brachioradialis, extensor carpi radialis longus and brevis, extensor digitorum, extensor digiti minimi, extensor carpi ulnaris, Supinator, abductor pollicis longus, extensor pollicis brevis, extensor pollicis longus, extensor indicis |  |  |  |  |  |  |

***Lower Limb***

| **Limb** | **Compart-ment** | **Muscle** | **Fatty infiltration score**  **(0-5)** | | **Atrophy score**  **(0-3)** | | **Inflammation score**  **(0-5, A/B)** | |
| --- | --- | --- | --- | --- | --- | --- | --- | --- |
|  |  |  | R | L | R | L | R | L |
| Pelvic | Hip extensors | Gluteus maximus |  |  |  |  |  |  |
|  |  | Gluteus medius |  |  |  |  |  |  |
|  |  | Gluteus minimus |  |  |  |  |  |  |
|  |  | Tensor fascia latae |  |  |  |  |  |  |
|  | Hip rotators / flexors | Obturator externus |  |  |  |  |  |  |
|  |  | Obturator internus |  |  |  |  |  |  |
|  |  | Pectineus |  |  |  |  |  |  |
| Lower limb - Thigh | Anterior (quads) | Rectus femoris |  |  |  |  |  |  |
|  |  | Vastus medialis |  |  |  |  |  |  |
|  |  | Vastus intermedius |  |  |  |  |  |  |
|  |  | Vastus lateralis |  |  |  |  |  |  |
|  | Medial  (adductors) | Sartorius |  |  |  |  |  |  |
|  |  | Gracilis |  |  |  |  |  |  |
|  |  | Adductor longus |  |  |  |  |  |  |
|  |  | Adductor brevis |  |  |  |  |  |  |
|  |  | Adductor magnus |  |  |  |  |  |  |
|  | Posterior (hamstrings) | Semi-membranosus |  |  |  |  |  |  |
|  |  | Semi-tendinosus |  |  |  |  |  |  |
|  |  | *Long* head biceps femoris |  |  |  |  |  |  |
|  |  | *Short* head biceps femoris |  |  |  |  |  |  |
| Lower limb – Calf | Anterior/ Lateral | Tibialis anterior |  |  |  |  |  |  |
|  |  | Ext. digitorum and hallucis longus |  |  |  |  |  |  |
|  |  | Peroneus (fibularis) group  -longus/brevis |  |  |  |  |  |  |
|  | Posterior | Flexor digitorum longus |  |  |  |  |  |  |
|  |  | Tibialis posterior |  |  |  |  |  |  |
|  |  | Soleus |  |  |  |  |  |  |
|  |  | *Lateral* gastrocnemius |  |  |  |  |  |  |
|  |  | *Medial* gastrocnemius |  |  |  |  |  |  |

# Study inclusion/exclusion criteria

Inclusion Criteria:

- Adults (>18 years)
- Able to give full informed consent
- A diagnosis of IBM or PM according to agreed diagnostic criteria (A Bohan & Peter, 1975; Anthony Bohan & Peter, 1975; Lloyd et al., 2014)

Exclusion Criteria:

- Any contraindication to MRI
- Any contraindication to amyloid-PET/CT imaging
- Pregnancy, breastfeeding or planning to become pregnant within 1 month of participating in the study
- Women of childbearing potential that do not use any form of contraception

For the PM cohort we restricted recruitment to those aged >45 years. We also retrospectively applied the ACR/EULAR (International Myositis Classification Criteria Project) IIM classification criteria to the PM group, with all cases meeting the minimum probability cut-off of 75%.(Lundberg et al., 2017)

# References

Bohan, A., & Peter, J. B. (1975). Polymyositis and dermatomyositis (first of two parts). *The New England Journal of Medicine*, *292*(7), 344–347. https://doi.org/10.1056/NEJM197502132920706

Bohan, A., & Peter, J. B. (1975). Polymyositis and dermatomyositis (second of two parts). *The New England Journal of Medicine*, *292*(8), 403–407. https://doi.org/10.1056/NEJM197502202920807

Lloyd, T. E., Mammen, A. L., Amato, A. a, Weiss, M. D., Needham, M., & Greenberg, S. a. (2014). Evaluation and construction of diagnostic criteria for inclusion body myositis. *Neurology*, *83*(5), 426–433. https://doi.org/10.1212/WNL.0000000000000642

Lundberg, I. E., Tjärnlund, A., Bottai, M., Werth, V. P., Pilkington, C., de Visser, M., … International Myositis Classification Criteria Project Consortium, the Euromyositis Register, and the J. D. C. B. S. and R. (UK and I. (2017). 2017 European League Against Rheumatism/American College of Rheumatology Classification Criteria for Adult and Juvenile Idiopathic Inflammatory Myopathies and Their Major Subgroups. *Arthritis & Rheumatology (Hoboken, N.J.)*, *69*(12), 2271–2282. https://doi.org/10.1002/art.40320

Mercuri, E., Talim, B., Moghadaszadeh, B., Petit, N., Brockington, M., Counsell, S., … Merlini, L. (2002). Clinical and imaging findings in six cases of congenital muscular dystrophy with rigid spine syndrome linked to chromosome 1p (RSMD1). *Neuromuscular Disorders : NMD*, *12*(7-8), 631–638. Retrieved from http://www.ncbi.nlm.nih.gov/pubmed/12207930

Yao, L., & Gai, N. (2012). Fat-corrected T2 measurement as a marker of active muscle disease in inflammatory myopathy. *AJR. American Journal of Roentgenology*, *198*(5), W475–W481. https://doi.org/10.2214/AJR.11.7113

Yushkevich, P. A., Piven, J., Hazlett, H. C., Smith, R. G., Ho, S., Gee, J. C., & Gerig, G. (2006). User-guided 3D active contour segmentation of anatomical structures: Significantly improved efficiency and reliability. *NeuroImage*, *31*(3), 1116–1128. https://doi.org/10.1016/j.neuroimage.2006.01.015
